# Analysis of risk factors for recurrence in infertile endometrial cancer patients after *in vitro* fertilization treatment

**DOI:** 10.3389/fendo.2023.1224622

**Published:** 2023-11-22

**Authors:** Hongyi Wei, Ningning Pan, Yang Wang, Caihong Ma

**Affiliations:** ^1^ Center for Reproductive Medicine, Department of Obstetrics and Gynecology, Peking University Third Hospital, Beijing, China; ^2^ Department of Obstetrics and Gynecology, Peking University International Hospital, Beijing, China; ^3^ National Clinical Research Center for Obstetrics and Gynecology, Peking University Third Hospital, Beijing, China; ^4^ Key Laboratory of Assisted Reproduction, Ministry of Education, Peking University, Beijing, China; ^5^ Beijing Key Laboratory of Reproductive Endocrinology and Assisted Reproductive Technology, Peking University Third Hospital, Beijing, China

**Keywords:** endometrial cancer, *in vitro* fertilization, assisted reproductive technology, fertility preservation, recurrence, controlled ovarian stimulation

## Abstract

**Purpose:**

To investigate the oncologic outcomes of patients with early-stage endometrioid endometrial cancer (EEC) treated with *in vitro* fertilization/intracytoplasmic sperm injection (IVF/ICSI) following fertility-sparing treatment (FST).

**Methods:**

A total of 62 patients who underwent IVF/ICSI treatment in a single fertility center between June 2010 and December 2021 after conservative treatment for early-stage EEC were assigned to a recurrence group and a non-recurrence group. Their clinical characteristics and disease outcomes were retrospectively evaluated.

**Results:**

The 62 women with complete remission (CR) after FST for EEC underwent 103 IVF cycles, resulting in 41 fresh embryo transfers (ETs) and 70 frozen–thawed transfers; 27 (43.55%) achieved clinical pregnancies, and 20 (32.26%) gave birth to a total of 23 live neonates. Additionally, nine patients had live births from natural pregnancies after IVF failure, bringing the cumulative live birth rate to 46.77% (29/62). After a median follow-up period of 53.88 months (range 20.2–127.5 months), 17 patients (27.42%) experienced recurrence within 2.8 to 57.9 months after the first controlled ovarian stimulation (COS). The probability of relapse at 1, 2, and 3 years after the initiation of COS was 14.52% (9/62), 21% (13/62), and 25.81% (16/62), respectively. Factors such as the time to CR, the time to IVF, the frequency of COS, maintenance treatment before IVF, and histology type were not found to significantly affect recurrence (*p* = 0.079, 0.182, 0.093, 0.267, and 0.41, respectively). Live births (hazard ratio (HR): 0.28, 95% CI: 0.082–0.962, *p* = 0.043) and the protocol of letrozole plus gonadotropin-releasing hormone (GnRH) antagonist/agonist used during IVF (HR: 0.1, 95% CI: 0.011–0.882, *p* = 0.038) were identified as independent favorable factors for recurrence.

**Conclusions:**

Live birth was associated with decreased recurrence of EEC. Reducing estrogen levels during COS may serve to mitigate the risk of endometrial cancer recurrence.

## Introduction

Endometrioid endometrial cancer (EEC) is a frequently occurring malignancy within the realm of gynecologic oncology. Approximately 7%–8% of EEC cases are diagnosed in women of reproductive age. Conservative treatment with hormone therapy could be considered a feasible approach to maintain fertility in women diagnosed with early-stage, grade 1 endometrial adenocarcinoma and has shown a remission rate between 76.2% and 81.4% (1). Given the high recurrence rate of EEC after complete remission (24%–40%), it is recommended that they conceive as soon as possible after the successful remission of endometrial cancer, and if necessary, assisted reproductive technology (ART) should be actively accepted to improve the pregnancy rate. Nevertheless, a major concern for clinicians is that the controlled ovarian stimulation (COS) procedure used for fertility treatment can result in high estrogen levels, which has the potential to increase the recurrence risk in patients with EEC or atypical endometrial hyperplasia (AEH). According to the study by Vaugon et al., there was no statistically significant difference in the probability of 2-year recurrence between women who underwent *in vitro* fertilization (IVF) treatment and those who did not, with recurrence rates of 37.7% and 55.7%, respectively (2). As compared to those with AEH, patients with EEC had a poorer live birth rate after ART (odds ratio (OR): 3.14, 95% CI: 1.07–9.18) and a higher recurrence rate (OR: 4.94, 95% CI: 2.41–10.15) (3, 4). There is an urgent need for clinical research aimed at reducing the risk of recurrence while improving pregnancy rates in patients with EEC who are at higher risk of relapse during IVF treatment. The purpose of this study was to assess the safety of IVF treatment in patients with EEC, as well as identify the factors that may impact the risk of recurrence.

## Materials and methods

This study protocol was approved by the Ethics Committee of the PUTH (No. M2022572). We performed a retrospective analysis of a prospectively managed database of infertility patients with early-stage EEC who achieved complete response after fertility-sparing treatment and subsequently underwent IVF–embryo transfer (ET) treatment at the Reproductive Center of Peking University Third Hospital between June 2010 and December 2021. The histologic diagnosis of EEC and response to the treatment were made based on hysteroscopic biopsy or dilation and curettage (D&C) after necessary examinations to rule out myometrial or cervical invasion and distant metastases. The patients were administered oral progestins [megestrol acetate (MA) or medroxyprogesterone acetate (MPA)] with or without gonadotropin-releasing hormone (GnRH) agonist or insertion of levonorgestrel-releasing intrauterine device (LNG-IUD) according to standard International Federation of Gynecology and Obstetrics (FIGO) guidelines. In the histologic interpretation of treatment response, complete remission was defined as a condition with no cancerous or hyperplastic lesions.

Once patients achieved complete remission (CR), they were encouraged to conceive at the earliest possible time. Patients with infertility were referred to reproductive endocrinology and infertility (REI) specialists directly for ART. The time to CR was calculated as the interval from the start of conservative treatment to CR. The time to IVF was defined as the interval between CR and the first IVF cycle. Patients were followed up until April 1, 2023.

Experienced physicians selected the suitable COS protocol, taking into account a holistic assessment of each patient’s baseline hormone levels, age, body mass index (BMI), antral follicle count (AFC), medication adherence, and financial feasibility. Mainly four COS protocols were performed in this study, including the GnRH agonist and antagonist protocols, letrozole+GnRH antagonist/agonist protocol, and progestin-primed ovarian stimulation (PPOS). GnRH agonist and antagonist protocols were utilized, along with recombinant follicle-stimulating hormone (FSH) or urinary human menopausal gonadotropin in the COS process. The initial dose of gonadotropin (Gn) was determined based on the patient’s ovarian reserve (150–300 U/day) and adjusted as necessary during COS. The GnRH long agonist protocol involved administering 0.1 mg of short-acting GnRH agonist during the luteal phase of the previous menstrual cycle, followed by starting Gn 14 days later until the trigger day. Once serum E_2_ is >1,500 pmol/L, letrozole may be added at a dosage of 2.5 or 5.0 mg/day until the trigger day. In the GnRH antagonist protocol, Gn was initiated on the second or third day of the menstrual cycle, and when the leading follicle diameter reached 12–14 mm, a daily injection of 0.25 mg of GnRH antagonist was introduced until the trigger day. In the letrozole/antagonist protocol, patients orally received letrozole at a dosage of 2.5 or 5.0 mg/day from the second day of the menstrual cycle for 5 days within the GnRH antagonist protocol. Ovulation trigger was achieved by administering human chorionic gonadotropin (hCG) (6,500 IU) or GnRH agonist (0.2 mg) combined with 2,000 IU hCG when at least two follicles reached a diameter greater than 18 mm. Oocyte retrieval was performed 34 to 38 hours later. Oocytes were fertilized using conventional IVF or intracytoplasmic sperm injection. The development and quality of embryos were assessed on day 3 (5). Blastocyst morphology was evaluated on day 5 or 6 using the Gardner grading system (6). Embryo transfer was conducted with only one or two embryos implanted per patient at a time. After embryo transfer, regular luteal support was provided with oral, vaginal, or intramuscular progesterone per day from the day of ET to the 10th week of gestation. In case of an increased risk for ovarian hyperstimulation syndrome (OHSS), thin endometrium, or some other reasons, a “freeze-all” strategy was performed for some patients. Subsequent frozen–thawed transfer was performed on day 3 or 5 through a natural cycle, ovulation induction cycle, or artificial cycle. The natural cycle protocol and the ovulation induction protocol are both categorized as non-artificial cycle protocols.

The total Gn use was the sum of the Gn used in each COS cycle. E_2_ peak during COS was defined as the highest estrogen level value in the COS cycles. Clinical pregnancy was diagnosed by ultrasonographic visualization of the intrauterine gestational sac after embryo transfer. Live birth was defined as the delivery of any viable infant at 24 weeks or more of gestation.

### Statistical methods

The data were analyzed using SPSS version 26 (SPSS Inc., Chicago, IL, USA). Data are presented as mean ± SD for continuous variables with normal distribution and were compared using t-test. Non-normally distributed continuous data are presented as median (range) and were compared using the Mann–Whitney U-test. Categorical data are presented as number (percentage) and were compared using the χ^2^ test or Fisher’s exact probability test. The Cox regression model was used for correlation analysis of cancer relapse. The Kaplan–Meier method was utilized to calculate recurrence rates, and the log-rank test was employed to compare the differences in these rates between groups. *p*-Value <0.05 was regarded as statistically significant.

## Results

### Baseline characteristics

Up to April 1, 2023, 17 out of 62 (27.42%) patients experienced recurrence during a mean follow-up of 57.31 ± 30.11 months (median 53.88, range 20.20 to 127.50 months). The mean time from COS to relapse was 16.47 ± 13.63 months (median 12.4, range from 2.8 to 57.9 months). The probability of relapse at 1, 2, and 3 years after initiation of COS was 14.52% (9/62), 21% (13/62), and 25.81% (16/62), respectively.

As shown in **Table 1**, the mean age at diagnosis was 31.31 ± 4.40 years; 55 (88.71%) patients were primarily infertile. Of the 62 women, 56 (90.3%) had an initial pathological diagnosis of grade 1, and six (9.7%) patients were diagnosed with grade 2. After cancer diagnosis, 51 (82.3%) patients were treated with oral progestins (MPA or MA), and the other patients were treated with oral progestins in combination with LNG-IUD or GnRH agonist. The mean number of D&C was 4.84 ± 1.83 times, and all the women responded to treatment with a mean time to CR of 8.85 ± 6.02 months. The median time interval between CR and IVF was 4.87 (0.57–54.23) months. Patients were assigned to the recurrence (17 cases) and non-recurrence (45 cases) groups. There were no significant differences in age, BMI, histology, medical comorbidity, basal sex hormone levels, AFC, conservative treatment regimens, time to IVF, and maintenance therapy between the recurrence group and the non-recurrence group. The duration of disease remission in patients with recurrence was longer than that in the non-recurrence group; however, the difference was not significant (11.03 ± 8.67 *vs.* 8.02 ± 4.57, *p* = 0.079).

**Table 1:** Characteristics of baseline and conservative treatment in the analysis cohort.

		Total n=62	Non-recurrence group n=45	Recurrence group n= 17	*P* value
Age at diagnosis (y) , mean±SD		31.31±4.40	31.18±4.65	31.65±3.74	0.711
	< 35	42 (67.7)	32 (71.1)	10 (58.8)	0.356
	≥35	20 (32.3)	13 (28.9)	7 (41.2)	
BMI (kg/m^2^) , mean±SD		26.34±4.49	26.13±4.10	26.88±5.52	0.564
	<30	48 (77.4)	34 (75.6)	14 (82.4)	0.739
	≥30	14 (22.6)	11 (24.4)	3 (17.6)	
Primary infertility, n (%)		55 (88.7)	39 (86.7)	16 (94.1)	0.408
Histology, n (%)					
	grade 1	56 (90.3)	42 (93.3)	14 (82.4)	0.410
	grade2	6 (9.7)	3 (6.7)	3 (17.6)	
Duration of infertility (y), mean±SD		3.47±2.66	3.13±2.63	4.35±2.62	0.108
Basal AFC, mean±SD		11.56±8.59	11.42±8.84	11.94±8.14	0.834
AMH (ng/mL), median (range)		1.54 (0.06-17.48)	1.49 (0.06-17.48)	2.40 (0.2-9.89)	0.287
Hypertension, n (%)		9 (14.5)	6 (13.3)	3 (17.6)	0.696
Diabetes, n (%)		2 (3.2)	0 (0.0)	2 (11.8)	0.125
PCOS, n (%)		13(21.0)	7 (15.6)	6 (35.3)	0.176
No.of D&C procedures, mean±SD		4.84±1.83	4.67±1.51	5.29±2.49	0.342
Treatment regimen, n (%)					
	Oral progestin	51 (82.3)	37 (82.2)	14 (82.4)	1.00
	Oral progestin+LNG-IUD/GnRHa	11 (17.7)	8 (17.8)	3 (17.6)	
Time to CR (mo), mean±SD		8.85±6.02	8.02±4.57	11.03±8.67	0.079
	≤9, n (%)	41 (66.1)	30 (66.7)	11 (64.7)	0.884
	>9, n (%)	21 (33.9)	15 (33.3)	6 (35.3)	
Tme to IVF (mo), median (range)		4.87 (0.57-54.23)	5.13 (0.57-54.23)	4.28 (1.13-25.43)	0.182
Maintenance therapy before IVF, n (%)		54 (87.1)	41 (91.1)	13 (76.5)	0.267
Follow-up period after CR(mo) , mean±SD		50.51±25.60	45.98±25.35	65.46±21.21	0.041*

SD, standard deviation; BMI, body mass index; AFC, antral follicle count; AMH, anti-mullerian hormone; PCOS, polycystic ovary syndrome; D&C, dilation and curettage; LNG-IUD, levonorgestrel-releasing intrauterine device; GnRHa, gonadotropin-releasing hormone agonist; CR, complete remission; IVF, in vitro fertilization.

*P<0.05.

### Stimulation characteristics and outcomes

As shown in **Table 2**, 62 patients underwent a total of 103 IVF/intracytoplasmic sperm injection (ICSI) cycles, resulting in 111 embryo transfers, including 41 fresh ETs and 70 frozen–thawed ETs. The average COS cycles started and the ET cycles performed in each patient were 1.67 ± 1.08 and 2.0 (0.00–11.0), respectively. The recurrence group consisted of 17 patients who underwent 34 COS cycles, while the non-recurrence group consisted of 45 patients who underwent 69 COS cycles. Patients with recurrence experienced a higher number of COS cycles compared to the non-recurrence group, although the difference was not statistically significant (2.10 ± 1.52 *vs.* 1.55 ± 0.90, *p* = 0.093). Mainly four COS protocols were used in this study, including the GnRH agonist protocol, GnRH antagonist protocol, letrozole+GnRH antagonist/agonist protocol, and PPOS. Thirteen patients used only the GnRH agonist protocol, 17 patients used only the GnRH antagonist protocol, 15 patients employed letrozole+GnRH antagonist/agonist protocols, two patients used only the PPOS protocol, and 15 patients used more than two different protocols during IVF. Patients who used only the letrozole+GnRH antagonist/agonist protocol had a significantly lower recurrence rate than those who used other stimulation protocols (*p* = 0.048). There were no significant differences in LNG-IUD used during COS, total gonadotropin used, serum E_2_ level on the trigger day, number of oocytes and transferable embryos retrieved, endometrium thickness, and the time interval between IVF and first ET between the two groups. Among the 62 patients who underwent IVF at our center, 20 patients achieved live births, and nine patients conceived spontaneously and had live births after IVF-ET failure. Patients who experienced relapse had a significantly decreased live birth rate of 23.5%, compared to those without relapse, who had a live birth rate of 55.6% (*p* = 0.024). Patients with recurrence exhibited a substantially higher hysterectomy rate of 58.8%, compared to those without recurrence, who had a hysterectomy rate of 4.4% (*p* < 0.001). Among the 17 patients with recurrence, nine received conservative retreatment with oral progestins or LNG-IUD plus GnRH agonist, eight underwent hysterectomy due to relapse in five patients and no further desire for pregnancy attempts in three patients; they are now disease-free survivors. Of the nine patients who had retreatment, five were still undergoing therapy, one achieved CR and was receiving treatment for thin endometrium, two achieved CR and underwent hysterectomy after a successful birth with one patient experiencing relapse again, and one achieved CR and had a successful birth with close monitoring.

**Table 2:** Characteristics of COS and embryo transfer in the analysis cohort.

Characteristics		Total n = 62	Non-recurrence group n = 45	Recurrence group n = 17	*P*-value
Age at IVF (years), mean ± SD		32.50 ± 4.10	32.44 ± 4.22	32.65 ± 3.87	0.864
No. of COS cycles, n		103	69	34	
No. of COS per patient, mean ± SD		1.67 ± 1.08	1.55 ± 0.90	2.10 ± 1.52	0.093
	1, n (%)	33 (53.2)	27 (60)	6 (35.3)	0.082
	2, n (%)	23 (37.1)	15 (33.3)	8 (47.1)	0.318
	>2, n (%)	6 (9.7)	3 (6.7)	3 (17.6)	0.333
COS protocols, n (%)					
	GnRH agonist	13 (21.0)	10 (22.2)	3 (17.6)	0.964
	GnRH antagonist	17 (27.4)	11 (24.4)	6 (35.3)	0.393
	Letrozole+GnRH antagonist/agonist	15 (24.2)	14 (31.1)	1 (5.9)	0.048*
	PPOS	2 (3.2)	2 (4.4)	0	1.00
	Mixed protocols	15 (24.2)	8 (19.0)	7 (35.0)	0.113
Using LNG-IUD during COS, n (%)		16 (25.8)	13 (28.9)	3 (17.6)	0.564
Total gonadotropin use (IU), mean ± SD		4,198.51 ± 2,919.13	3,921.56 ± 2,845.58	4,931.62 ± 3,071.31	0.227
E_2_ peak during COS, median (range)		4,259.50 (503–37,481)	4,372.00 (503.00–37,481.00)	4,147.00 (536.00–11,608.00)	0.943
No. of oocytes retrieved per patient, mean ± SD		14.84 ± 11.22	14.56 ± 12.42	15.59 ± 7.41	0.691
No. of transferable embryos per patient, mean ± SD		6.23 ± 5.04	5.87 ± 5.30	7.18 ± 4.26	0.366
Endometrium on the day of hCG injection or luteal support start (mm), mean ± SD		8.15 ± 1.87	8.233 ± 1.72	7.91 ± 2.28	0.551
Time interval between IVF and first ET (days), median (range)		87.5 (12–1,573)	105 (12–1,573)	59 (12–509)	0.458
Patients with ET cancellation, n (%)		9 (14.5)	8 (17.8)	1 (5.9)	0.434
No. of ET cycles, n		111	76	35	
	Fresh ET, n (%)	41 (36.94)	24 (31.6)	17 (48.6)	0.085
	Frozen–thawed ET, n (%)	70 (63.06)	52 (68.4)	18 (51.4)	
Patients with frozen–thawed ET, n (%)		38 (61.3)	25 (55.6)	13 (76.5)	0.131
	Artificial cycle	15 (39.5)	10 (66.7)	5 (33.3)	0.927
	Non-artificial cycle	20 (52.6)	13 (65.0)	7 (35.0)	0.914
	Mixed cycles	3 (7.9)	2 (66.7)	1 (33.3)	1.000
No. of ET per patient, median (range)		2.0 (0.00–11.0)	1.00 (0.00–11)	2.00 (1.00–4.00)	0.449
Transferred good quality embryos per patient, median (range)		2.00 (0.00–16.00)	2.00 (0.00–16.00)	2.00 (0.00–8.00)	0.284
With a live birth, n (%)		29 (46.8)	25 (55.6)	4 (23.5)	0.024*
	Conceived with IVF	20 (32.3)	17 (37.8)	3 (17.6)	0.130
	Conceived spontaneously	9 (14.5)	8 (17.8)	1 (5.9)	0.236
Hysterectomy, n (%)		12 (17.74)	2 (4.4)	10 (58.8)	<0.001*
	For recurrence	6	0	6	
	After successful birth	1	0	1	
	No longer desire to experience pregnancy	5	2	3	

IVF, in vitro fertilization; SD, standard deviation; COS, controlled ovarian stimulation; PPOS, progestin-primed ovarian stimulation; LNG-IUD, levonorgestrel-releasing intrauterine device; E_2_: estradiol; ET, embryo transfer.

*p < 0.05.

### Affected factors associated with relapse after IVF cycles

**Figure 1:**
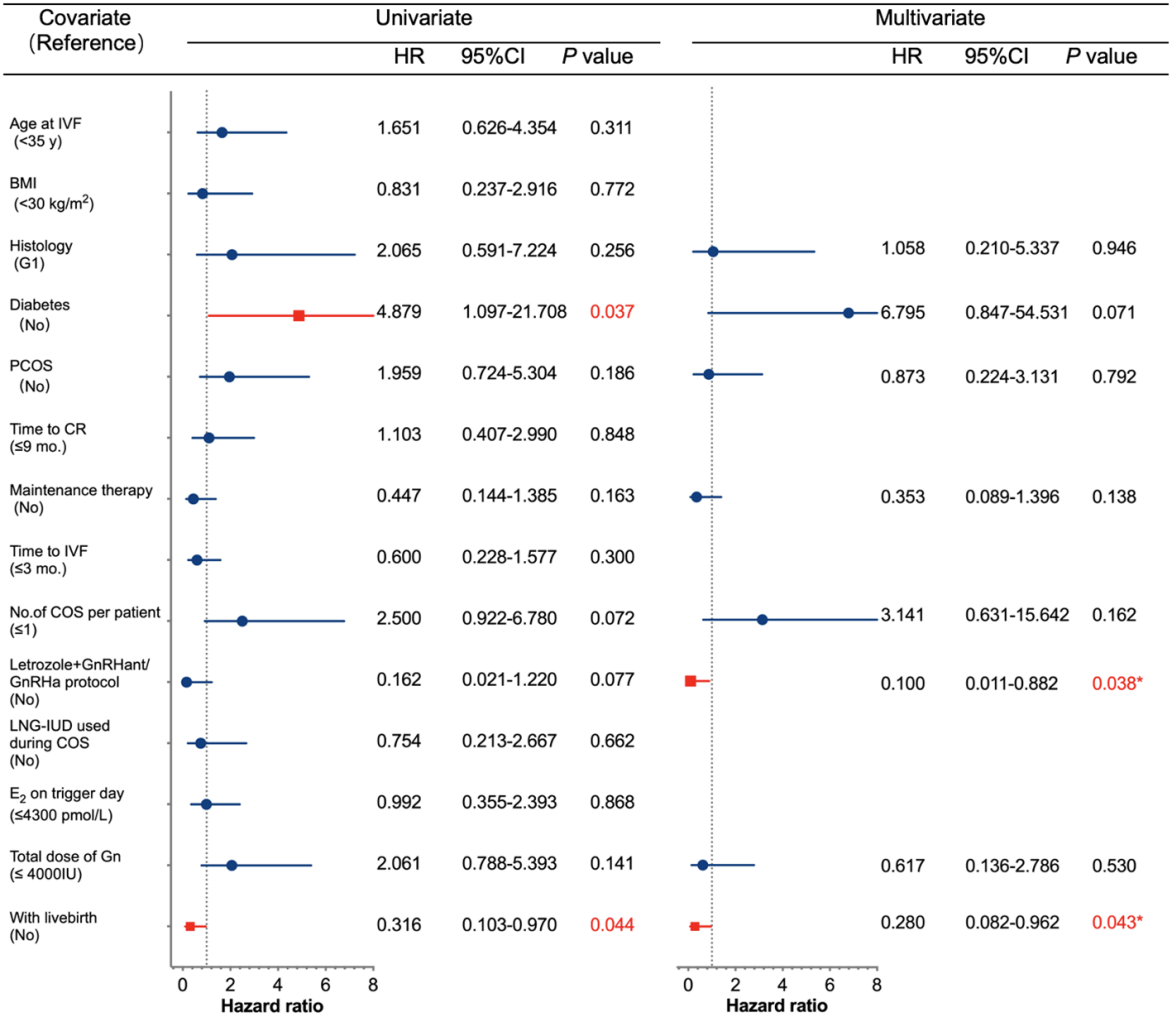
Univariate and multivariate Cox regression analyses of factors associated with recurrence. IVF, *in vitro* fertilization; BMI, body mass index; G1, grade 1; PCOS, polycystic ovary syndrome; CR, complete remission; COS, controlled ovarian stimulation; GnRHant, GnRH antagonist: GRHa, GnRH agonist; LNG-IUD, levonorgestrel-releasing intrauterine device; E_2_, estradiol; Gn, gonadotropin; CI, confidence interval; HR, hazard ratio.

**Figure 2:**
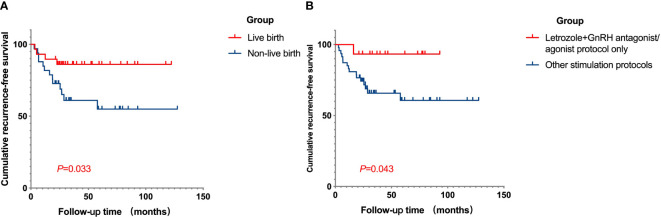
Cumulative RFS curves in fertility-sparing EEC patients after IVF/ICSI treatment. **(A)** The cumulative RFS in patients of live birth group and non-live birth group. The RFS was longer in patients who successfully had a child compared to those who were unable to conceive. **(B)** The cumulative RFS for patients who underwent COS with letrozole plus GnRH antagonist/agonist protocol only, or not. The cumulative RFS was longer for patients who used the letrozole plus GnRH antagonist/agonist protocol only for ovulation induction. RFS, recurrence-free survival; EEC, early-stage endometrial cancer; IVF/ICSI, *in vitro* fertilization/intracytoplasmic sperm injection; COS, controlled ovarian stimulation.

Several potential high-risk factors impacting the oncological outcomes were further analyzed through multivariate regression (**Figure 1**). The Cox univariate analysis demonstrated that age, BMI, histology, polycystic ovary syndrome (PCOS), time to complete remission, the interval between complete remission and the first IVF, LNG-IUD insertion, and maintenance therapy were not related to disease relapse. Diabetes was a risk factor for tumor recurrence (hazard ratio (HR): 4.879, 95% CI: 1.097–21.708, *p* = 0.037), while successful live birth was a protective factor for tumor recurrence (HR: 0.316, 95% CI: 0.103–0.970, *p* = 0.044). Variables with *p* < 0.3 from the univariate analysis results (histology (*p* = 0.256), diabetes (*p* = 0.037), PCOS (*p* = 0.186), maintenance therapy (*p* = 0.163), number of COS cycles per patient (*p* = 0.072), letrozole+GnRH antagonist/agonist protocol (*p* = 0.077), total Gn use (*p* = 0.141), and with a live birth (*p* = 0.044)) were further incorporated into a Cox multivariate regression model. The findings indicated that the letrozole+GnRH antagonist/agonist protocol and successful live birth were independently associated with a decreased risk of EEC recurrence after IVF (HR: 0.1, 95% CI: 0.011–0.882, *p* = 0.038, HR: 0.28, 95% CI: 0.082–0.962, *p* = 0.043, respectively). The impact of live births and protocols on recurrence-free survival (RFS) for EEC is depicted in **Figure 2**.

## Discussion

This study revealed that the overall recurrence rate for patients with EEC after IVF treatment was 27.42% over a median follow-up period of 53.88 months (ranging from 20.2 to 127.5 months). The average time from IVF treatment to relapse was 16.47 ± 13.63 months (median 12.4, ranging from 2.8 to 57.9 months). Research by Gallos et al. showed that patients with EEC had a pooled relapse rate of 40.6% (95% CI: 33.1%–49.8%) (1), whereas the recurrence rate for those who received ART treatment was reported to be 21.0%–47.0% with a median recurrence time of 12–28 months (1, 7, 8), which was consistent with our reports on recurrence in EEC patients after IVF treatment. After achieving complete remission of the disease, the majority of patients (87.1%) received maintenance therapy with medication before IVF treatment to prevent recurrence. Most of these patients underwent IVF treatment for fertility assistance approximately 4 months after disease remission.

In order to further elucidate the factors that lead to disease recurrence in endometrial cancer patients undergoing IVF treatment, we assigned the patients into a recurrence group and a non-recurrence group. Ovarian stimulation used in IVF treatment can lead to a hyperestrogenic state, which may contribute to the progression or recurrence of hormone-responsive tumors such as endometrial cancer. Therefore, parameters such as the number of stimulation cycles, the peak serum estradiol concentrations during stimulation, the COS protocol type, the total dose of gonadotropin administered, and the endometrial thickness are considered the most relevant factors to monitor. We found that patients who employed a COS protocol combining letrozole with GnRH antagonist or agonist had a lower recurrence rate of 5.9% compared to other COS protocols (31.1%). Multivariate regression analysis showed that this protocol had a protective effect in preventing recurrence (HR: 0.1, 95% CI: 0.011–0.882, *p* = 0.038). Letrozole is a highly selective, non-steroidal oral aromatase inhibitor that can inhibit the conversion of testosterone to estradiol and androstenedione to estrone in a reversible manner. Kawahara and colleagues proposed that the utilization of aromatase inhibitors during ovarian stimulation could inhibit the proliferation of uterine endometrial cancer in a xenograft mouse model (9). Oktay et al. reported that the use of letrozole in stimulation for IVF resulted in comparable outcomes for breast cancer patients and infertility patients who were subjected to IVF using standard protocols (10). A Cochrane review in 2017, which included 3,599 participants, showed that there was no clear evidence that letrozole with or without gonadotropins resulted in different outcomes compared to gonadotropins alone for both the general population and poor responders undergoing IVF treatment (11). However, using letrozole during COS may offer several benefits, including reducing the required dose of gonadotropins, lowering the risk of high estrogen exposure, and potentially providing a more patient-friendly and endometrium-protective approach. It may be an effective strategy for IVF in patients with EEC.

More ovulation stimulation cycles may lead to more frequent hyperestrogenic states, which is speculated to possibly promote tumor recurrence, especially in patients with endometrial cancer. The current study found that the recurrence rate for patients with more than one COS cycle was slightly higher than that for patients with a single COS cycle, but the differences were not significant (*p* = 0.082), which is consistent with previous research findings (2, 4, 12).

Frozen–thawed ET is generally considered a safe option for women who have had endometrial tumors, but the potential effects of different endometrial preparation methods on endometrial tumor relapse during FET should be cause for caution. Using a natural menstrual cycle without hormonal stimulation may be associated with a lower risk of endometrial tumor relapse. This method avoids exogenous hormones that could potentially stimulate residual cancer cells. However, it may not be suitable for all patients, especially those with irregular menstrual cycles and thin endometrium. The artificial cycle involves using exogenous hormones (estrogen and progesterone) to prepare the endometrium. While this method is effective for endometrial preparation, it may raise concerns about whether the hormonal environment increases the risk of tumor relapse or progression. In the current study, we did not find an increased risk, and long-term follow-up data may be needed.

Numerous studies have investigated the association between pregnancy and tumor recurrence, consistently reporting a significantly lower rate of recurrence in patients who have experienced live births compared to those who have not. Prior research by Ushijima et al. indicated that women who had experienced at least one pregnancy had a significantly lower risk of recurrence compared to those who had never been pregnant (13). In our study involving 62 patients, we also found that patients who did not have live births exhibited a significantly higher recurrence rate than those who had live births (35.14% *vs.* 16.0%, *p* = 0.024). One possible explanation could be the heightened levels of progesterone during pregnancy, coupled with the complete detachment of the decidua from the uterus after delivery. Furthermore, pregnant women with conditions such as obesity and PCOS may be able to avoid single exposure to estrogen for certain periods, potentially delaying tumor recurrence and progression. However, this proposed mechanism, suggesting that pregnancy can prevent the recurrence of EEC/AEH lesions, remains speculative as no studies have conclusively verified it to date.

Fertility-sparing treatment for EEC patients with G2 is not recommended in most guidelines. However, it is applied in selected cases after providing evidence-based counseling, leading to a shared decision-making process with the patient. A meta-analysis involving 84 patients with grade 2 endometrial cancer showed that 20 of them relapsed at varying intervals ranging from 6 to 142 months post-hormonal treatment (14). In this study, six patients with G2 underwent IVF treatment, with three patients experiencing relapse after IVF treatment. More research is needed to clarify the safety and effectiveness of IVF in patients with G2.

Recurrence of endometrial cancer is not an absolute contraindication for preserving fertility. It is still necessary to combine the patient’s wishes, indications for fertility preservation, and the response to previous treatment to develop a conservative treatment plan. After recurrence for EEC patients, retreatment can still achieve a relatively high remission rate (66.7%–100%), and the pregnancy rate after retreatment has no significant difference compared to that after the initial treatment (20.8%–29%) (15–17). Of the 17 patients who experienced recurrence in this study, nine were re-treated conservatively with oral progestins or LNG-IUD in combination with GnRH agonists. Two achieved complete remission and opted for hysterectomy after fulfilling their childbearing desires, with one of them experiencing a second recurrence. Hysterectomy is recommended for patients who had successful birth, as the cause of endometrial cancer remains unclear, and despite treatment and fertility completion, the underlying cause persists, leaving a risk for tumor recurrence and metastasis.

As far as we know, this study is one of the most extensive investigations to concentrate on the effects and consequences of IVF/ICSI treatment on patients diagnosed with EEC at a single center. Selection bias may be the limitation of a single-center retrospective study. This study included the patients whose lesions were confined to the endometrium. The influence of superficial myometrial infiltration or lesion size during initial treatment on tumor recurrence after IVF treatment requires further investigation.

## Conclusions

Our study showed that live birth may be associated with decreased relapse. Letrozole as an adjunct in GnRH antagonist/agonist cycles during controlled ovarian stimulation serves as a protective factor against the recurrence of endometrial cancer. Reducing estrogen levels during COS may potentially mitigate the risk of endometrial cancer recurrence.

## Data availability statement

The original contributions presented in the study are included in the article/supplementary material. Further inquiries can be directed to the corresponding author.

## Ethics statement

The studies involving humans were approved by Peking University Third Hospital Research Ethics Committee. The studies were conducted in accordance with the local legislation and institutional requirements. The participants provided their written informed consent to participate in this study.

## Author contributions

CM and HW contributed to conception and design of the study. HW and NP organized the database. HW and YW performed the statistical analysis. HW wrote the first draft of the manuscript and CM revised the manuscript. All authors contributed to manuscript revision, read, and approved the submitted version.
